# Austere life-courses and foreclosed futures: A relational geographical approach to work, housing, and family across austerity Europe

**DOI:** 10.1177/20438206251316005

**Published:** 2025-02-04

**Authors:** Santiago del Río, Sarah Marie Hall, Elizabeth Ackerley, Laura Fenton

**Affiliations:** 5292 University of Manchester, UK; 5292 University of Manchester, UK; 5292 University of Manchester, UK; 5292 University of Manchester, UK

**Keywords:** Austerity, family, foreclosed futures, home, life-course, work, young people

## Abstract

This article seeks to advance geographical understandings of the impact of austerity on young adults in Europe and particularly on their social, relational, and temporal sense of the future. We adopt a life-course perspective to theorise personal, generational, institutional, and social change in relation to one another. Firstly, we progress relational life-course perspectives in human geography to illustrate how place-based experiences of austerity shape lived experiences, temporalities, and normative ideas surrounding life transitions. We introduce the concept of ‘foreclosed futures’ to re-examine conceptualisations of young adults’ eroding material conditions as a postponement of adulthood. We argue that the enduring impact of austerity contests the theorisation of young adults’ experiences of precarity as a transient elongation of youth. Expanding upon non-teleological life-course perspectives, we show that while austere institutions have foreclosed stable futures, the paths to precarity are myriad and rooted in geographically varied forms of austerity. Secondly, in examining how austere institutions shape young people's work, housing, and family biographies, we argue that, rather than leading to de-institutionalisation, austerity marks a process of life-course re-institutionalisation or ‘familialisation of the life-course’. To conclude, this article proposes a series of prompts for future international empirical research on austere life-courses and foreclosed futures.

## Introduction

Austerity has altered the life-courses of many young adults in Europe, and globally influencing the life decisions and futures they can make, imagine, and envision (Hall, 2023). Expanding upon relational approaches in human geography (Dyck, 2005; Lawson, 2007; Massey, 2004), this article develops a geographical perspective to examine the life-courses of austerity and its futures. We introduce the concept of *foreclosed futures* to shed light on how, for many young people, the roads to everyday and future stability have been denied. Generations of young Europeans have grown up under austerity, their lives shaped by socio-political changes that will influence their futures long after austerity policies have lapsed (Hall, 2022). Young people have been shown to heavily feel the impacts of austerity policies, in part given their greater exposure to housing and work precarity and underfunded public services and institutions (see Barford and Gray, 2022; Eurofound, 2024, Horton et al., 2022). In this context, the incremental generalisation of work insecurity and precarity across Europe has led to theories positing that transitions to adulthood have been protracted and delayed (Arnett, 2014; Cote, 2000; Furlong, 2007; Galland, 2003). While these ideas help capture changes in transitions to adulthood, they can have the effect of causally connecting the achievement of economic stability with adulthood, and at the same time, conflate the temporary and transitional nature of youth with precarity.

With the contextual focus of Europe and an emphasis on three key facets of daily life – housing, work, and family – we argue that the enduring effects of neoliberalism and austerity demand a shift away from traditional notions of adulthood as the attainment of stability linked to post-war patterns of work, housing, and family formation. Austerity is a socio-economic policy marked by the reduction of public spending, implemented as a political response to address fiscal deficits and national debt in the aftermath of the Global Financial Crisis (Kitson et al., 2011). In effect, austerity shifted the economic burden of the Global Financial Crisis onto ordinary people with devastating social consequences. Expanding on this approach, we hold onto established feminist geography arguments on austerity as both a political-economic and personal condition, affecting relationships, identities, emotions, hopes, and mental and physical health (see Hall, 2019; Hitchen and Raynor, 2019). To deepen this understanding, rather than reducing parts of the paper to theorising the specificities of place-based contexts, we instead draw on extensive literature from across Europe to illuminate how austerity seeps into all aspects of everyday life in enduring ways, reshaping life-course norms, temporalities, dreams, and expectations. In doing so, in the second part of the article, we examine key socio-economic trends exacerbated by austerity, such as housing unaffordability, the growing influence of intergenerational wealth, in-work poverty, underemployment, and the decline of formal care systems. We illustrate how both geography and rising intersectional inequalities intensified the lived effects of these phenomena.

From a range of perspectives, life-course scholars have argued that the rise of neoliberalism and the loosening of Fordist structures have individualised the life-course, making contemporary life more fluid and unpredictable (Beck, 1992; Cote, 2000; Woodman and Wyn, 2015). In this sense, the idea of the ‘de-institutionalisation’ of the life-course (Henretta, 1994; Settersten, 2003b) describes the dwindling role of social institutions in shaping biographies. Re-examining the suitability of ‘de-institutionalisation’, we show how austere social institutions increasingly shape life decisions and social reproduction strategies in direct and indirect ways. Through the idea of *famililisation of the life-course*, we build upon feminist geography to highlight how austerity is an institutionalised political project based on the transfer of social reproduction responsibilities to gendered interdependencies of families, friends, communities, and the third sector (Daskalaki et al., 2021; Hall, 2019; Jupp, 2017).

Against the backdrop of austerity, this article contributes to ongoing and vibrant discussions on life-course theories within feminist and social geographies (Holdsworth and Hall, 2022; Hörschelmann, 2011; Katz and Monk, 1993; Skelton, 2017), presenting new avenues for conceptual and empirical scholarship. We draw on conceptualisations of austerity as a process that is relationally, spatially and temporally diverse, sometimes manifesting as an unending condition rather than a transitional phase (Hall, 2019). Within this framework, we introduce the novel notion of *foreclosed futures* by building on the Cambridge Dictionary's dual definition of ‘foreclose’ – to take possession and to prevent – to signify how austerity has seised control over young people's futures while imposing constraints that inhibit their ability to envision and achieve stable and secure futures. We use ‘foreclosed futures’ to indicate that while the roads to stability are increasingly blocked, the paths to precarity are fluid and geographically contingent.

Amid an increasingly shared reality of normalised precarity (see Furlong et al., 2017), ‘foreclosed futures’ can provide a deeper understanding of how the impact of austerity on path-dependent housing and labour regimes, welfare systems and localised intersectional inequalities shape young people's expectation aspirations and dreams. For instance, youth underemployment is pervasive across Europe (Roberts, 2008), but in southern Europe, youth unemployment takes on a more prominent role (Eurofound, 2024). Homeownership is increasingly inaccessible for young people, with rental prices sharply rising (Ronald and Arundel, 2023). While this is a common trend, tenant rights vary widely across Europe, with tenants being notably vulnerable in England and enjoying more protection in Germany (Kettunen and Ruonavaara, 2021). Similarly, though it is well-documented that austerity has disproportionally impacted young people (Eurofound, 2024), the severity of welfare cuts (Taylor-Gooby et al., 2017), the age at which young adults leave the parental home, fertility rates, and the average age for first-time parenthood reveal differences across European contexts (Eusostat, 2022).

This article therefore has two key aims. Firstly, to advance a relational life-course perspective that captures how young adults’ lived experiences of austerity have reshaped their life trajectories, temporalities, expectations, and normative ideas of life transitions. And secondly, through the concept of ‘foreclosed futures’ – amid a common tendency towards housing unaffordability, the increasing role of intergenerational wealth, in-work poverty, underemployment, and the decline of formal care systems across Europe – we aim to spark dialogue that sheds light on how different forms of austerity shape young people's perceptions and expectations about the future, both materially and emotionally. In the first part of the article, we show that the enduring presence of austerity and precarity calls into question established notions of transitions as delayed or prolonged (Arnett, 2014; Cote, 2000; Furlong, 2007; Galland, 2003). We argue that the rootedness of these ideas in the post-war temporalities and norms has led to the conflation of precarity – a permanent condition for many – with youth, a transitional phase. At the same time, we contest theses that argue the life-course has become de-institutionalised to demonstrate that austerity creates its own kind of life-course institutionalisation. The second part of the article draws on and advances relational perspectives of the life-course to analyse the impact of austerity in work, housing, and family biographies. To close, we seek to inspire future international research by proposing a series of prompts that seek to progress empirical studies on austere life-courses and foreclosed futures.

## Critical life-course perspectives: Decentring post-war adulthood norms

The life-course perspective, rooted in Thomas and Znaniecki's ‘life history’, Mannheim's use of ‘generation’ and Parsons’ ‘age differentiation’ was systematically developed in the 1960s (see Bernardi et al., 2019) to study the organisation of lives within given social, cultural, and institutional arrangements. This perspective was advanced to study interconnected age-graded trajectories linked to aspects such as work careers, family pathways, and life transitions, and how they are influenced by changing conditions and future possibilities (Elder, 1994). In Western society, the life-course has traditionally been conceptualised as a structured sequence of age-based transitions punctuated by three periods encompassing an early segment dedicated to early education and work training, a mid-phase of continuous work, and a later phase focused on leisure and retirement (Settersten, 2003b: 81). We posit that tracing back to these early beginnings of life-course theories can hold the key to understanding their current uses and limitations.

A lineal sense of life-course development is commonly associated with a process of ‘standardisation’ of the life-course that emerged in the post-war period (Settersten, 2003a). In Western industrialised societies, with variations shaped by path-dependent welfare regimes, the advent of Fordist mass production and an institutional framework supporting a gendered division of labour created normative notions of adulthood as comprised of independent living, economic stability, and nuclear family formation (see Mayer, 2004). Reflecting this, life-course perspectives were originally framed around the idea of ‘standardised’ and ‘institutionalised’ life patterns (Kohli et al., 1986; Settersten, 2003b) – a form of life-course normativity that, although unevenly realised, served as an aspirational ideal in the post-war period.

From the 1980s, it is argued, the loosening of Fordist social structures ‘de-standardised’, ‘de-institutionalised’ and ‘individualised’ life-courses, making them more fluid and unpredictable in relation to normative post-war life-courses (Beck, 1992; Henretta, 1994; Settersten, 2003a). This thesis emphasises that private lives and family structures have undergone a pluralistic transformation, which along with increased instability in work careers, has given rise to ever more flexible life-courses. While we recognise that adaptation and flux in theories of the life-course may indeed reflect real-life changes, these binaries of standardisation/de-standardisation and institutionalisation/de-institutionalisation are too coherent. On the one hand, the post-war notions of ‘standardisation’ can be misleading as they universalise the experience of male industrial workers in this period without accounting for geographical variations and gender and racial exclusions. On the other, references to individualisation and fluidity, rooted in the idea of de-institutionalisation, do not fully capture the specific ways in which neoliberal, and more recently, austerity policies ‘re-institutionalise’ the life-course, particularly within the institution of the family.

While key contributions acknowledge that precarity has always existed not only in early life but also in adulthood and late life (Katz, 2020; Phillipson, 2020), as Blatterer notes (2007) ‘adulthood’ is generally conceptualised as a signifier of independence, autonomy and stability. And yet, these normative notions of adulthood did not and do not reflect the reality of many people. Feminist scholars have extensively shown that, in the post-war period, married women were institutionally constructed as dependents who could only access direct and social incomes through their status as wives (see Abramovitz, 2017). Similarly, LGBTQ+ people were considered a distinct economic class who were not allowed to constitute contractual families – the institution through which mortgages, inheritance, and spousal pensions were accessed (Butler, 1997). More importantly, this normative life-course model overlooks the reality of much of the world, where waged employment and the nuclear family are not as predominant. In most Global South countries, extended families and informal labour are more common, playing a central role in daily life (Mezzadri, 2019). In other words, the institutionalised, standardised life-course of the post-war period and the predictability, stability, and security linked to this were only relevant for a relatively small part of the world's population and reinforced racial, classed, and heteronormative hierarchies. In this sense, the idea of post-war ‘standardisation’ that has dominated the life-course literature must be understood against its geographical limits and constitutive exclusions.

Current notions of adulthood, even when transitions to adulthood are argued to be delayed, anachronistically refer to the status of a particular group of workers in a brief historical moment in a small number of countries. Some scholars have worked to develop these ideas, advancing critical perspectives of the life-course. Neilson and Rossiter (2008) note that when observing the history of capitalism from a historical and geographical perspective, the ontological security associated with Fordist societies is a fleeting exception rather than the norm. Similarly, Schwartz and Flynn (2023: 34) consider the variegated post-war housing regimes, where lower housing costs relative to incomes were achieved through diverse strategies such as the expansion of social housing, rent regulation, and state-backed mortgage financing, to be a historical exception. They contend that the current reality of housing unaffordability represents a ‘partial regression to social patterns typifying the pre-war era’. In what follows, we build on these contributions to argue that references to prolonged youth and delayed adulthood continue to universalise patterns of work, housing, and family formation that do not reflect everyday life under austerity.

Departing from post-war notions of standardisation, a sociological body of literature (Blatterer, 2007; Leccardi, 2012; Woodman and Wyn, 2015) argues that adulthood should no longer be understood as a stable identity. Blatterer notes (2007) that contemporary adulthood is increasingly defined by fluidity and flexibility as opposed to stability and ‘settling down’. Similarly, Woodman and Wyn (2015: 90) note that we can no longer understand ‘youth as experimentation leading to a stable adult identity’ as ‘new youth’ ‘involves messy and incremental steps into a ‘new adulthood’ that itself is increasingly defined by a precariousness and relative instability’. However, beyond disembedded references to ‘new youth’ and ‘new adulthood’ as ‘messy’, ‘fluid’, ‘flexible’, and ‘precarious’, we show that relational perspectives in human geography (Dyck, 2005; Lawson, 2007; Massey, 2004) can support a rigorous characterisation of contextually varied precarities, foreclosed paths to future stability and the lived experiences and temporalities stemming from this. The next sections develop these ideas further.

### Foreclosed futures: Delayed adulthood or enduring precarity?

Temporality is a key conceptual anchor in life-course approaches. Life-course literature has traditionally studied the temporality of transitions through notions such as ‘timely’ and ‘untimely transitions’ (Elder et al., 2003). Untimely transitions are associated with a minority of people who are exposed to greater risks due to limited support from social institutions. Hence, untimeliness is linked to the constrained choices of a non-normative minority which faces the risks of not having the institutional support backing ‘standardised’ life-courses (Elder et al., 2003). Under austerity such ideas about temporalities that demarcate adequate moments of ‘transition’ do not capture the everyday experiences of most young people in Europe (Hörschelmann, 2011). This is because the once-expected markers of adulthood (financial, social, and housing independence), as prescribed by post-war, and still present in post-Fordist narratives and people's aspirations, have become largely unattainable to a growing number of people; and yet theories of the life-course are yet to ‘keep up’ with these lived realities.

Today, young people across Europe increasingly move from moments of employment and unemployment, change jobs across different sectors, relocate to cities and countries searching for better opportunities, continue their education and/or retrain while they work, live in shared accommodation for extended periods, constantly move houses, and return to their parent's home after having experienced independent living (Wilkinson and Ortega-Alcazar, 2019). In many cases, these dynamics exceed the age boundaries that have conventionally been associated with ‘youth’. This phenomenon has been characterised as an elongation of youth and postponement of outright adulthood through terms such as ‘prolonged youth’ (Galland, 2003), the ‘protraction of youth’ (Furlong, 2007), ‘arrested adulthood’ (Cote, 2000), and ‘emerging adulthood’ (Arnett, 2014), thus retaining core traditional ideas within life transitions theory. These ideas suggest that not a minority, but a large majority of precarious young and not-so-young adults are ‘off-time’. This paradoxical proposition is only tenable because ‘adulthood’ continues to be considered a fixed destination to which most young people arrive, albeit later in life.

While these ideas acknowledge the processual nature of generational change, they also suggest that precarity is a transitional phase that concludes upon the achievement of outright adulthood. This in turn entails that leaving precarity behind is a precondition to attaining adulthood. Perhaps inadvertently, this logic reproduces teleological notions of the life-course by conflating youth with precarity and security with adulthood. Here, adulthood continues to act as a signifier of independence, achievement, stability, and ultimate destination after a prolonged, yet transient, period of economic difficulties. Given that precarity is increasingly pervasive and persistent, and that one can enter, leave, and return to poverty or instability across time (Hall, 2022), there is a need for relational and geographical perspectives that shed light on the non-linear temporalities of austere life-courses.

The persistence of post-war notions of adulthood paradoxically suggests that for some people, particularly those economically disadvantaged, ‘youth’ (by definition a transitional period) extends perpetually. This framework suggests that people in their late 30s or older who are unemployed or underemployed and still live with their parent(s) or in shared accommodation have not yet achieved the status of a ‘full adult’. By contrast, someone in their late 20s with a permanent, well-remunerated job, living independently would be considered an outright adult. The persistence of teleological notions of transitions is thus denying adulthood to people who have been denied economic security. In this sense, current conceptualisations of transitions as delayed or prolonged continue to fail to represent the reality of many people, especially those experiencing the enduring impacts of austerity. As we shown in the exploring austere life-courses through a relational geographical lens section, this is not to say that, at a societal level, normative notions of transitions do not hold sway (Blatterer, 2007), rather they often manifest as increasingly inaccessible aspirations or ‘foreclosed futures’.

While the post-war notions of transitions to adulthood identified here are largely exclusionary in terms of geography, race, gender, and sexuality, the delayed or protracted transitions argument overlooks the significance of social class. To explain, it has been extensively documented that the post-2008 regimes of austerity have heightened young adults’ reliance on their parents (a further intergenerational support) to attain economic security and independence (Adkins et al., 2020; Ronald and Arundel, 2023). Insecurity, precarity, intergenerational cohabitation, and a trend towards generational downward mobility, undoubtedly foster frustration and negatively affect people's notions and sense of self (Pimlott-Wilson, 2017). However, this often has very little to do with personal delays or individual failures to secure economic stability (Skelton, 2017). Instead, these phenomena must be contextualised within a framework of intersectional class dynamics and geopolitical inequalities. Here, rather than ‘delayed adulthood’, we pose our notion of ‘foreclosed futures’ to illuminate how paths to stability are increasingly denied reshaping life-course norms, temporalities, and expectations.

As we examine in the exploring austere life-courses through a relational geographical lens section, higher rates of young adults living with their parents, youth underemployment and in-work poverty (Eurostat, 2022) are not the result of a delayed achievement of adulthood. Instead, they are the outcome of long-standing geographical inequalities, the ideological orientation of variegated welfare regimes, the impacts of austerity on housing and work, and the context-specific social reproduction strategies mobilised against these circumstances. As we now go on to reveal, despite the emphasis on ‘individualisation’ and ‘de-institutionalisation’, austerity policies and austere institutions intentionally and by design transfer reproductive responsibilities to intergenerational families and non-government institutions.

### The institutionalised familialisation of austere life-courses

As shown above, the rise and hegemony of neoliberalism are linked to a process of ‘de-institutionalisation’ and ‘individualisation’ which are used to explain greater fluidity and unpredictability in life-courses. From the 1990s, sociologists refer to greater heterogeneity in the life-course while emphasising more individual agency through terms such as ‘do-it-yourself biographies’ and ‘choice biographies’ (Beck, 1992). These terms highlight how biographies are increasingly shaped by individual choices due to the diminishing influence of institutions in shaping life trajectories. In connecting the emergence of neoliberalism with the formation of ‘risk societies’, Beck (1992) contends that the loosening of social structures supporting the post-war standardised life-course – a phenomenon characterised as ‘de-institutionalisation’ (Settersten, 2003b) or ‘institutionalised individualism’ (Beck and Beck-Gernsheim, 2002) – means that faced with unpredictable circumstances, individuals must become more flexible, agile and self-reliant.

Notions of the life-course that conceptualise ‘individualisation’ as greater agency (Giddens, 1991) have been duly criticised for undermining how growing inequality and social constraints limit the range of life decisions that one can make or even imagine (Dawson, 2012). However, the idea of ‘de-institutionalisation’ (narrowly understood as the withdrawal of the state from social reproduction), has not garnered as much critical scrutiny as ‘individualisation’. In engaging with this debate, Riley and Riley Jr. (1994) argued that there was a structural lag between new ways of life and the social institutions supporting them, implying that it was just a matter of time for policy to ‘catch up’ with social change. In this period, the idea of de-institutionalisation faced scrutiny with critics arguing that a process of re-institutionalisation was underway. This process was either described as the re-institution of a new welfare architecture based on a new social contract (Pierson, 2001) or the rise of a new form of welfare capitalism (Gilbert et al., 1990) in which the state actively supports the increasing role of non-government welfare institutions in social reproduction.

However, the ideas of de-institutionalisation or re-institutionalisation – when understood as the emergence of a new social contract – fail to present and articulate neoliberalism and austerity as classed institutional projects that are consciously geared towards the production of unequal and precarious conjunctures (Hall and Massey, 2010). The emphasis on de-institutionalisation as mere welfare state retrenchment emerges from a misleading critique of neoliberalism as a form of laissez-faire capitalism based on self-regulating markets, non-state intervention, and dwindling social institutions. As Slobodian (2018) and Bruff and Tansel (2019) adeptly explain, neoliberals and the neoliberal project did not advocate for the disappearance of state and supranational institutions. Far from that, neoliberals granted social institutions a central role not to unleash unfettered markets but to encase them so that they can facilitate upward redistribution. Similarly, the idea of ‘re-institutionalisation’ as a lag between societal change and adapting welfare institutions omits that, instead of catching up with a reality foreign to them, neoliberal government institutions designed and engendered the precarious versions of flexibilisation, individualisation, and unending austerity that characterise contemporary life-courses in Europe.

In this vein, departing from the idea of ‘institutionalised individualism’, understood as a process in which instead of the family, ‘the individual is becoming the basic unit of social reproduction for the first time in history’ (Beck and Beck-Gernsheim, 2002: 22), feminist thinkers have rightly pointed out that the neoliberal ethos of self-responsibility, exacerbated by austerity, is not so much based on an offloading of reproductive responsibilities from the state and capital onto self-reliant individuals. Instead, social reproduction responsibilities have been transferred to gendered networks of interdependency such as families, friends, communities, and third-sector organisations (Hall, 2019; Jupp, 2017). Indeed, as Wendy Brown (2019: 37) shows, the variegated implementation of the neoliberal project is not only rooted in the ‘entrepreneurialisation’ and ‘de-proletarianisation’ of workers but also in re-grounding social reproduction in practices of familial self-provisioning. In this line, Cooper (2017) has shown that in the 1970s the neoliberal project was posed against the advances of emancipatory social movements that demanded the expansion and universalisation of the welfare state beyond the family. As such, it can be argued that life-courses under austerity have not only become individualised but more importantly *familialised*.

Far from having become de-institutionalised, the life-course is shaped by austere institutions and policies that influence the life-course in a negative way (Leisering, 2003), that is by transferring the formation of the life-course to the family. Therefore, we argue that it is more appropriate to refer to the *institutionalised familialisation of the life-course* rather than ‘individualisation’, ‘de-institutionalisation’, or ‘institutionalised individualism’. As we detail in the exploring austere life-courses through a relational geographical lens section, austerity has made economic security and independent living increasingly dependent on the intergenerational family and the constitution of two-earner families. A policy regime conducive to asset appreciation, wage stagnation and family-based forms of welfare (Adkins et al., 2020) means that those who cannot rely on any family support and/or do not wish to form a nuclear family tend to be poorer. The assetisation and familialisation of welfare exacerbates challenges for historically marginalised groups such as the medium and lower strata of the working-class, queer people who are rejected by their families, single people, one-earner families with dependents (disproportionally headed by women), and working-class migrants who, instead of getting support from their transnational families, often need to support them.

Departing from ideas that positively emphasise individualisation as more choice, we argue that the diversification of how one can be precarious does not constitute an advancement of contemporary lives. While precarious work, housing and family pathways have been diversified, the roads to stability have been foreclosed. To gain a better understanding of these lived experiences and the social institutions shaping them, we think that there is a necessity for research that examines not only how austerity constrains young people's life-courses, but also how young people personally experience and navigate the material discrepancy between neoliberal discourses on autonomy and self-reliance and the persistence of austere material conditions. In what follows, we make the case for theoretical and empirical foci that could help to achieve this goal.

## Exploring austere life-courses through a relational geographical lens

We now turn our focus to exploring how austere life-courses, as described in the previous sections, can be further articulated and investigated through a relational lens. We argue that relational approaches to the life-course complement a conceptualisation of life transitions as non-linear trajectories. Influential writings across human geography (Hörschelmann, 2011; Katz and Monk, 1993; Skelton, 2017) call for further exploration of a relational notion of the life-course that recognises diversity and contingency while questioning teleological notions of human development. Hörschelmann (2011, p. 379) in particular argues that the traditional treatment of life transitions is ‘well ordered and safe, adopting a staged chronology that does not account for delayed, multiple, reverse and uncertain transitions that are worked through in the everyday lives of individuals and that can be moments of both crisis and opportunity’. This perspective enables an understanding of adulthood, not as merely a delayed phenomenon, but as a fluid process contingent on geography, class, race, gender, sexuality, residence status, and disability (see also Barron, 2021; Brown et al., 2012; Hopkins and Pain, 2007)

We build on these approaches to progress a non-teleological perspective on transitions that depart from lineal routes and fixed origins/destinations while scrutinising the complexity of disparate forms of everyday life (see Holdsworth and Hall, 2022). As a burgeoning research topic and with growing relationally comparative studies (see Davies, 2023; Tulumello, 2023; van Lanen, 2023), there is an appetite for research exploring how varied austerity regimes shape young people's biographies. As noted above, contemporary life-courses are characterised as increasingly unpredictable and fluid. However, under austerity, housing insecurity, labour precarity, and protracted family formation in Europe have become often predictable (in some cases, permanent) features of the life-course, foreclosing futures.

To explore the life-course through a relational geographical lens, we draw on conceptualisations that position austerity as a condition temporally varied that ‘can ebb and flow into everyday lives, and it can feel unending’ (Hall, 2022: para. 3). This perspective recognises that austere social institutions shape everyday life in enduring and diverse ways, effects that persist long after austerity policies and politics. Furthermore, our perspective on ‘austere life-courses and foreclosed futures’ seeks to bring together relational comparisons examining varied forms of austerity across Europe (Bailey et al., 2021; Davies, 2023; Kitson et al., 2011) with feminist geographies of austerity and everyday life (Hall, 2019; Hitchen and Raynor, 2019; Hughes and Valentine, 2010; Tarrant and Hall, 2020) to shed light on how austerity reshapes life-course norms, temporalities, aspirations, expectations, and emotional landscapes. This approach can deepen understanding of how varied labour and housing regimes, path-dependent welfare systems, and place-based intersectional inequalities enable and constrain how futures can be made and imagined. In what follows, we engage with general socio-economic tendencies across Europe, which stem from or have been accelerated by austerity, to identify changes and continuities in young people's housing, work, and family biographies (and the links between them). We pose a series of prompts that aim to animate further qualitative, place-based empirical research illuminating the life-courses of austerity.

### Insecure homes, unachievable assets

While the politico-economic shifts leading to the current housing crisis have been extensively analysed (Aalbers, 2008; Wijburg et al., 2018) and there is a growing body of research that explores young adults’ lived experiences of housing insecurity (van Lanen, 2022; Wilkinson and Ortega-Alcázar, 2019), our approach to the life-course can illuminate how young adults emotionally and materially imagine their housing futures and how this affects their expectations and normative ideas of transitions and their temporalities. Housing is perhaps the most compelling case of how austerity has altered the life-course. Across Europe, the decimation of social housing (Rolnik, 2013), the liberalisation and financialisation of rent (Wijburg et al., 2018), and the ongoing appreciation of assets vis a-vis real wages (Adkins et al., 2020) support an ideology (Ronald, 2009) that produces mortgaged homeownership as the only economically viable tenure form. Despite this, since 2008, homeownership has been denied to private tenants across Europe with private rent becoming the fastest-growing form of tenure in European large cities (Ronald and Arundel, 2023). Under these circumstances, many young adults have been condemned to rising, insecure rents in substandard houses, which are often shared with strangers (Wilkinson and Ortega-Alcázar, 2019).

Eurofound research (2024) finds a widening mismatch between aspiration and concrete plans in housing-related issues such as leaving parental home or moving in with a partner and homeownership. This gap between wishes and expectations is rooted in a European-wide tendency towards housing unaffordability and difficulties in accessing property wealth. Housing prices across the EU surged by 47% from 2010 to 2022, with significant increases in 24 Member States – Estonia, Lithuania, and Ireland saw the highest rent increases at 210%, 144%, and 84%, respectively (Eurofound, 2023). The lack of affordable housing has extended the average age at which young adults leave their parental home in Europe, rising from 26 to 28 between 2007 and 2019 (Eurofound, 2023). This trend is particularly pronounced in Spain, Croatia, Italy, Cyprus, Belgium, Greece, and Ireland. Recent research shows a significant decline in homeownership rates among the younger generation in European contexts with varied path-dependent trajectories, such as Germany, Hungary, Italy, Ireland, Spain, the United Kingdom, and Denmark (Flynn, 2020; Ronald and Arundel, 2023). Even in high-income countries with robust housing protections like the Netherlands, more households struggle to access property wealth, indicating that a strong job market position no longer guarantees access to homeownership (Arundel and Lennartz, 2020).

The so-called generation rent experiences non-linear housing paths, constantly moving between shared houses or returning to the family home (Arundel and Lennartz, 2017). This reality destabilises normative ideas of transitions to adulthood while hindering young adults’ ability to find the sense of permanency, belonging, and constancy traditionally linked to the idea of home. In Europe, house sharing has become a normalised living arrangement among young people (Arundel and Ronald, 2016). However, as Wilkinson and Ortega-Alcazar note (2019), the different circumstances that lead people to house sharing are the product of class, race, gender, and sexual dynamics (also see Bricocoli and Sabatinelli, 2016; Brown, 2015). The circumstances of a young professional who envisions house sharing as a temporary step before becoming a homeowner and who shares accommodation with friends of choice are very different from those who are trapped in unprotected rental markets. Intersectional approaches to house sharing demonstrate that sharing with strangers under precarious conditions has an impact on mental and physical health and increases the risk of being exposed to homophobia, transphobia, racism, and domestic violence (Wilkinson and Ortega-Alcázar, 2019)

Research demonstrates how, in diverse European contexts, the increasingly widespread phenomenon of ‘boomerang transitions’ – where individuals return to their parental homes after independent living – hinders expectations and aspirations for independence, disrupting the traditionally assumed linear progression into adulthood (Arundel and Lennartz, 2017; Berngruber, 2015; Tsekeris et al., 2017). However, this concept does not capture how this phenomenon, and austere living conditions more generally, may stem from different material conditions which are co-determined by class, race, gender, sexuality, and disability (Heath, 2008). Some people can go back to their parent's or childhood home after becoming homeless, due to partnership breakdown, to find temporary shelter while looking for a new home, to save up towards a housing deposit, or to care for family members (Wu and Grundy, 2023).

Similarly, in diverse contexts such as Spain, Czech Republic, Romania, Ireland, the Netherlands, and the United Kingdom (see Ronald and Arundel, 2023), the formation of two-earner couples and intergenerational support increasingly determines access to housing assets. The increasing impact of intergenerational wealth as a driver of inequality entails that variability in life-courses has more to do with intersectional class dynamics than personal choices (Hochstenbach, 2018). For instance, in the United Kingdom, being single dramatically increases the risk of becoming homeless, particularly among Black people and economic migrants (Jones and Pleace, 2010). In this line, it has been extensively documented (Ecker et al., 2019) that working-class LGBTQ+ people, particularly trans people, are more likely to become homeless, with family rejection being a key factor. In her study of the impact of sexual orientation on housing practices in Greece, Dagkouli-Kyriakoglou (2022), shows that increasing reliance on family welfare strongly determines strategic decisions around coming out.

Against this background, departing from ideas that refer to the de-institutionalisation of the life-course, we argue that austerity is both reinforcing and redefining family normativity and, in so doing, housing biographies. The political decisions and austerity policies – for example bank bailouts, asset relief programmes, low-interest rates, and quantitative easing – that restored housing asset inflation and financialisation and, at the same time, increased public debt to cement the ideological foundations for austerity may appear as disconnected from the privatisation of social reproduction within the family. However, these policies heavily determined the material conditions that shape life-courses.

House sharing, boomerang transitions, cohabitation with family members and (in some European contexts) private rent have traditionally been viewed as transitional living arrangements. In the context of austerity, many young adults living under these conditions surpass the ages typically associated with youth. The concept ‘foreclosed futures’, as outlined earlier, offers a foundation for research into young people's material and emotional lived experiences of housing might be influencing, and potentially altering, established understandings of life-courses and transitions. Are precarious housing arrangements perceived as temporary or permanent? Are they experienced as a trap from which one cannot escape or an obstacle in the path to stability? How do the ways in which this is emotionally experienced impact visions of housing futures, longings, aspirations, hopes, and life trajectories? How do geographical context, place-based housing regimes, race, gender, and sexuality shape housing presents and futures? We continue some of these themes below.

### Precarious work and dashed neoliberal dreams

As argued above, contemporary labour conditions run counter to the idea of stability as linked to a steady income and a linear career path. The sense of belonging, meanings, and promise of upward mobility linked to a coherent professional career have been disrupted (Sennett, 2006). The transition from a Fordist manufacturing-led economy to a so-called post-Fordist knowledge economy has been seconded by discourses that deem workers self-actualising human capital (Rose, 1990; Sennett, 2006). Under the neoliberal paradigm, constantly self-improved workers are encouraged to express their creativity, passions and lifestyles through work (Murgia and Poggio, 2014). Flexible workers are required to either update their skills to compete with an ever-more qualified working force for jobs below their qualification (MacDonald, 2011) or to innovate and take risks through entrepreneurial initiatives (Rose, 1990; Sennett, 2006). Under these circumstances, workers are made increasingly responsible for their present material conditions and futures (Skelton, 2017), which ideologically justifies the withdrawal of the state from social reproduction under the false claim that this unleashes individuals’ full potential.

Not only does this reformulation of work alter life-courses but also how the meanings linked to work cement (or undermine) notions of the self as well as ideas of progress and upward mobility. This is key to relational life-course thinking, which places selves and society in dialogue – including relational selves and identities across space-time. Paradoxically, neoliberal demands on young adults to become autonomous owners of their destiny take place in precarious labour markets in which underemployment has become the norm. Young adults increasingly experience non-linear professional pathways, drifting from one job to another, engaging in atypical forms of work, experiencing in-work poverty, periods of unemployment, and seeking alternative sources of income. In other words, while precarious work takes many different forms, permanent, well-remunerated employment is conspicuous by its absence. Supporting this, Furlong et al. (2017) argue that under neoliberalism precarious work has become the ‘new normal’, while arguing that it will only become ‘normalised’ when workers subjectively internalise this reality. Berry and McDaniel (2022) offer evidence of how austerity is accelerating the normalisation of precarity in the United Kingdom indicating that young people increasingly perceive precarious work not as a temporary phase but as an enduring aspect of their careers. This suggests that austerity is shaping future expectations and life-course norms.

In this line, research based on Eurostat data (van Lerven et al., 2022) shows that austerity policies have left European workers, on average, €3000 poorer annually, concluding that austerity entails a permanent loss rather than a delayed recovery. These losses disproportionally affect young people as evidenced by IMF research (Chen et al., 2018) which shows that the financial crisis significantly increased the risk of relative poverty among the youth compared to older demographics. After the 2008 crash, youth unemployment in several EU countries exceeded 40% (Eurofound, 2024), highlighting their vulnerability during downturns, with the EU27 you NEET (not in employment, education, or training) rate peaking at 16.1% in 2013, particularly affecting labour market re-integration for young women with children (O'Higgins and Brockie, 2024). In recent years, youth employment rates have shown a positive trend, and the NEET rate has reached a historic low. However, this improvement has not translated into better job quality, reinforcing a trend towards precarious work. Despite the rise in employment rates, poverty has persisted, shifting from out-of-work poverty to in-work poverty, particularly affecting low-educated, young, and racialised workers (Hiessl, 2020). European Commission research shows that in-work poverty has risen from 8.5% in 2008 to 9.4% in 2017 (Peña-Casas et al., 2019). While decreasing youth unemployment and NEET rates alleviate severe material deprivation, they have not sufficiently raised incomes to ensure a dignified standard of living.

An increase in non-standard forms of employment has been identified as one of the key factors in explaining in-work poverty (Peña-Casas et al., 2019). According to the European Council, in 2022, over 28.3 million people in the EU were employed by digital platforms, with numbers expected to rise to 43 million by 2025. In a context where austerity has intensified the neoliberal erosion of secure jobs, the expansion of app-based gig work, ‘mini-jobs’ in Germany, zero-hours contracts in the United Kingdom, and other forms of precarious work has proliferated (Rubery and Grimshaw, 2016). Evidence shows that workers in these jobs tend to be younger than average and are significantly paid below average (below the minimum wage for gig workers) (Datta et al., 2019; Wood et al., 2023). As Woodman (2013) argues, non-standard work schedules and irregular hours hinder young people's ability to coordinate time with friends and loved ones, harming their relationships.

In addition to in-work poverty, rising underemployment is particularly affecting young adults. The disjuncture between educational attainment and secure, well-remunerated employment is also key in understanding altered transitions to adulthood under austerity. One of the most important changes in the life-course in the last 40 years is the spectacular increase in university graduates. Going to university has become a normalised life milestone with more than 40% of the EU population aged 25–34 having attained a university degree (Eusostat, 2022). In increasingly precarious labour markets, this entails a discrepancy between the number of qualified workers and the supply of qualified labour. It is well established that the most defining characteristic of youth employment is the structural preponderance of underemployment not only in Europe but at a global scale (Roberts, 2008). The pervasive existence of youth underemployment means that graduates increasingly take low-paid jobs below their qualification, leaving non-graduates in an increasingly disadvantaged position (Furlong et al., 2017). While underemployment and in-work poverty are general problems across the board, in some Southern European regions, the youth unemployment rate still hovers above or near 40%,^1^ impacting young people's relationship with work, place, and future. Secular underemployment and unemployment can lead to all manner of results, including deteriorating mental health, burnout, reduced sociability, and compound problematic living relations (McDowell, 2012).

In exploring precarious work in Spain, the United Kingdom, and Italy, Murgia and Poggio (2014) have suggested the term ‘passion trap’ to characterise the tension between young people's material and emotional investment in finding jobs where they can express their passions and the demotivation linked to low- or no payment, instability or simply the impossibility of finding a job that matches their expectations. In this context, young adults have been pushed to seek alternative sources of income (Winkie, 2022) such as side jobs, subletting rooms, monetising their abilities in social media, investing in cryptocurrency, and app-based micro-trading. Some of these activities reproduce a neoliberal ethos of entrepreneurship, and yet they fail to foster promises of prosperity and individual success while extending the number of working hours necessary to meet bare needs.

As argued above, life-course literatures highlight that the work biographies of young people have become more individualised and unpredictable. However, it is reasonable to anticipate that generational downward mobility and precarity will remain persistent and normalised features in the lives of young and not-so-young adults. Sixteen years of austerity have seen a generation grow up in its shadow, making the ‘foreclosed futures’ perspective developed in this article a valuable lens for further empirical research into evolving relational geographies of austerity. This includes research which delves into how the discrepancy between the denial of stability and the broken promise of autonomy is lived and experienced. The post-war promise of upward mobility and stability has been shattered, while precarious labour markets materially diminish the discursive power of neoliberal discourses on self-responsibility. Nonetheless, post-war notions of stability and neoliberal ideas of individual success hold sway as aspirations at a discursive level. Against this background, qualitative research and relational comparison between different contexts can shed light on whether young adults attach new meanings, expectations, and aspirations to work as well as the emotional landscapes emerging from this. What kind of hopes and expectations do they place in work? Do they place their longings in neoliberal ideas of success? Do they play the ‘neoliberal game’ with scepticism? Do they seek alternatives and imagine futures that depart from both post-war and neoliberal notions of work? Or do all these contradictory attitudes converge creating polyhedric subject positions? How do young people experience precarity across space–time? Do they perceive it as a permanent condition? Do they blame themselves and if not, who or what? How do all these pressures affect young adults’ mental and physical health? These sentiments also hold for the next section regarding family decisions.

### Family choices and decisions in austere times

Intimate relationships, such as family decisions, may appear as a private matter disconnected from social institutions and capital redistribution. However, feminist thinkers have demonstrated that intimate relationships are shaped by classed interests, and the productive and reproductive systems emerging from this (Dalla Costa and James, 1975; Federici, 2012). The family has been theorised in multiple ways. Some sociological approaches to the family think about it in terms of family practices (Finch, 2007; Morgan, 2011; Neale and Smart, 1998) to emphasise that ‘family’ cannot be solely defined in terms of blood ties and legal arrangements. This approach highlights the agency of family members to re-signify family bonds, responsibilities and obligations. From a different perspective, feminist social reproduction theorists (Dalla Costa and James, 1975; Federici, 2012) conceptualise the family under capitalism as an institutional privatised system of social reproduction which acts as a subsidy to capital by regenerating labour power through unpaid or devalued reproductive work. Research within human geography has situated families within multi-scalar spatial and temporal change, arguing that everyday relationalities are fundamentally shaped by social and structural norms, inequalities and differences (Hughes and Valentine, 2010; Tarrant and Hall, 2020). Additionally, some scholars (Wilkinson, 2020) have noted that ‘family’ and ‘kinship’ are inherently exclusionary terms, which create boundaries between acceptable and non-acceptable intimate relationships.

Currently, there is a tension between the positive social recognition of non-traditional forms of family and the mobilisation of the family as a bounded entity of social reproduction that absorbs the shock of austerity (Hall, 2016). Coming back to earlier discussions in this paper, the idea of ‘individualisation’ of the life-course is often associated with the legal and social recognition of non-normative intimate relationships (Beck and Beck-Gernsheim, 2002). At the same time, ‘de-institutionalisation’ refers to the weakening of post-war redistributive policies and politics, which create conditions for more agency in family choices. Beck (2002: 97), the most influential advocate of these theses, argues that the family as, ‘a community of need held together by an obligation of solidarity’, is disappearing to be replaced by ‘the elective family’, which he defines as an ‘association of individual persons’ who face ‘different controls, risks and constraints’. Here, Beck implies that ‘de-institutionalisation’ and ‘individualisation’ endow people with a greater agency to make their own family choices. In doing so, he omits that, from the outset, neoliberalism and austerity are rooted in the familialisation of welfare and social care.

Like with ‘choice biographies’, terms such as ‘families of choice’ and ‘elective families’ obscure the economic constraints and the legal and policy frameworks that shape family choices under austerity. Far from being a spontaneous response to eroding conditions, feminist scholars such as Wendy Brown (2019) and Melinda Cooper (2017) have shown that the privatisation of social reproduction within the family is central to the neoliberal political and intellectual project from the outset. Departing from approaches that draw neat lines between the politics of recognition and the politics of redistribution, Cooper (2017) and Butler (1997) argue that identity politics and the politics of redistribution are always interconstitutive. For example, it is well established that post-war welfare supported and institutionalised the breadwinner model founded on a gendered division of labour and racial exclusions (Abramovitz, 2017). By contrast, a universal, non-means-tested welfare state providing free childcare, education, social care, disability support and old-age care would reinforce one's ability to engage in non-normative ways of life beyond the legal and biological family. Under austerity and financialisation, the attack on public services, precarious work, and the increasing costs of housing and other basic needs, on the one side, an asset appreciation and real wage stagnation, on the other, create conditions for the emergence of family-based forms of welfare (Adkins et al., 2020; Ronald and Arundel, 2023). Moreover, neoliberal policies of upward redistribution, intensified by austerity, have created material conditions for the rise of authoritarian, reactionary movements that seek to undermine the legal and social recognition of non-normative relationships. Currently, far-right governments and parties across Europe are pushing for a nostalgic revival of a reimagined patriarchal family structure while advancing anti-LGBTQ+ and anti-abortion agendas (Möser et al., 2021).

While people make their own decisions about intimate relationships, austerity restricts one's capacity to pursue, imagine and thrive under certain personal choices. The privatisation of social reproduction within the family constrains people's ability to pursue certain ways of life, such as living independently as a single person. For example, in the United Kingdom, single people pay a ‘single penalty’, resulting in a yearly shortfall of £10,000 (John, 2023). In this vein, some journalist pieces (Kambhampaty, 2022) have pointed out that housing precarity influences the anticipation of normative couple decisions such as moving in together or buying property. Housing unaffordability, and financial independence more generally, also impacts one's ability to leave violent and abusive relationships, disproportionally affecting women and children (also see Sanders-McDonagh et al., 2016). Ironically, despite the emphasis on ‘individualisation’ and ‘choice’, for many young adults, the only way to access housing stability via home ownership is to form a couple, pool two incomes and, in many cases, receive downward generational support from both sides of their respective families. Although family members engage in supportive strategies that stem from love, unconditionality, and moral obligations (Heath, 2008), when analysed at a societal level, internal family solidarity – for example supporting children's education, or intergenerational transfers – becomes a key factor in driving ‘compound inequality’ (Nunn and Tepe-Belfrage, 2019). In this sense, intergenerational transfers and the pooling of family incomes and wealth to acquire housing assets increasingly determine the divide between ontological security and permanent precarity.

Furthermore, feminist scholars have shown that the gendered moral economy of the family increasingly acts as an austerity shock absorber in diverse European contexts, such as the United Kingdom, Southern Europe and Nordic countries (Brown and Briguglio, 2022; Elomäki and Koskinen-Sandberg, 2020; Hall, 2016; Jupp, 2017). Cuts in government social spending, leading to the erosion or destruction of public sector jobs, disproportionately impact women, exacerbating gender inequalities in the labour market (López-Andreu and Rubery, 2021) while offloading reproductive responsibilities onto the home and the family. While a decline in formal care services has been particularly felt in the United Kingdom, Ireland, and the Southern European familialist welfare systems, research shows (Elomäki and Koskinen-Sandberg, 2020) a resurgence of women and naturalised caregivers in Nordic countries, historically distinguished by comprehensive public service provision. In these societies care is increasingly becoming more informal, family-based, marketized and performed by precarious migrant workers. In addition, the impact of austerity profoundly affects decisions such as those concerning having children, the number of children, and the timing of these choices. These decisions are not made in isolation but within a context of work precarity, increasing workloads, low pay, rising housing and childcare costs, decimation of care infrastructures, and reduced social support (Hall, 2023; Saunders, 2021). Consequences of the limiting of choice and decision are relational; they are at once personal and systemic impacting as they do on lived experience and demographic change. This is a good example of where a relational geographical approach is valuable, considering the impacts of austerity on life-courses as they play out across time and space.

The literature on European welfare systems has traditionally linked higher rates of inequality in southern Europe to familialist welfare systems (Allen et al., 2004; Ferrera, 1996). Under austerity, a tendency towards the familialisation of welfare in countries that did not traditionally have strong family contracts (see Adkins et al., 2020; Ronald and Arundel, 2023) must be read as both a cause and symptom of increasing social inequality. Against this context, how is the increasing economic role of the intergenerational family reshaping moral norms around family reciprocity and obligations in different contexts with different welfare traditions? How does the impact of austerity vary across contexts shaping normativity and future family imaginaries and decisions? How do young people experience the tensions between the pluralisation and recognition of non-normative intimate relationships and stringent material constraints pushing towards normative family arrangements? We now turn to summarise and conclude these discussions and outline our key contributions to relational geographies of austerity through the lens of ‘foreclosed futures’.

## Conclusion

This article has sought to advance and explicate a relational geographical approach to the life-course under austerity. With a focus on austere policies and conditions across Europe – where austerity policies have been implemented (and with varying and contextual idiosyncrasies) since the Global Economic Crisis of 2008. In delineating general socio-economic trends affecting young adults across Europe, we make the case for a geographical life-course perspective that sheds light on how multi-scalar spatial and temporal change seeps into everyday life. We argue that adopting a life-course perspective is one way to approach this task, which brings together and theorises personal, generational, institutional, and social change in relation to one another. We have argued that analysing austere life-courses relationally can illuminate how path-dependent social institutions, labour markets, housing systems, and everyday reproductive strategies interact with place-based austerity regimes, shaping people's everyday lives and futures.

Early in the article, through the idea of ‘foreclosed futures’ – which captures how austerity increasingly constrains paths towards stable futures – we lay out the key conceptual pieces of this argument. We posit that dominant ideas, such as that adulthood has become delayed and youth prolonged, are still rooted in post-war temporalities and norms. Building on these literatures, we contest long-standing assumptions about life transitions and markers of success, illustrating that normative concepts of the life-course need to be critically updated and pluralised to shed light on both the changing institutionalised systems that organise everyday life and the lived, affective responses to these changes. In the context of austerity, this includes revisiting notions of delayed futures to argue that categories of youth and adulthood are problematically positioned as congruent with financial dependence and independence, respectively. We recognise that post-Fordist life-course paradigms acknowledge diversity in the life-course. However, in expanding upon relational and geographical approaches to the life-course, we show how vague references to greater fluidity can be rooted in lived experiences of austerity that are shaped by path-dependent austere institutions. At the same time, we posit that ideas such as ‘individualisation’ and ‘de-institutionalisation’ miss that neoliberalism and austerity have produced institutionalised life-courses that, instead of enhancing individual agency and choice, reinforce the significance of the family; what we term the ‘familialisation of the life-course’. In this sense, beyond appreciated references to greater fluidity and plurality, we have shown how relational approaches to human geography can help characterise the diverse geographies of precarity and foreclosed stable futures.

After tracing and reframing these conceptual landscapes, we then presented ideas for how austere life-courses and foreclosed futures can be articulated through a relational geographical lens. Arranged according to three cross-cutting themes, loosely formulated around home, work, and family, we have drawn on ongoing research across Europe to demonstrate how thinking about austerity relationally and through the lifecourse offers new conceptual possibilities for future research. We highlight for example the need for further research exploring young people's everyday experiences of housing conditions, the hopes and expectations young people place in employment, and future family imaginaries in a context of economic precarity. This research agenda is vital to better understand the ways in which living in and through austerity shapes young people's everyday lives; past, present, and future.

We close now with some prompts that cut across the three original themes, aimed at those engaging in empirical investigation. How can, we ask, relational geographical concepts be ‘put to use’ for empirical investigation? What methodologies do we have at our disposal, and which might we need to develop future, in order to ‘get at’ multi-scalar change and continuity across and between life-courses? How can these methodologies shed light on the lived experiences, emotions, and temporalities of contingent austere life-courses? Who does such research involve, in what ways and with what consequences? And, lastly but importantly, how do we reckon with the mirroring of austere life-courses and foreclosed futures within our very institutions and disciplines, whilst at the same time engaging in meaningful, sensitive and exploratory research on these themes?

## Notes

1. Eurostat data.
